# An Explainable EEG-Based Human Activity Recognition Model Using Machine-Learning Approach and LIME

**DOI:** 10.3390/s23177452

**Published:** 2023-08-27

**Authors:** Iqram Hussain, Rafsan Jany, Richard Boyer, AKM Azad, Salem A. Alyami, Se Jin Park, Md Mehedi Hasan, Md Azam Hossain

**Affiliations:** 1 Department of Anesthesiology, Weill Cornell Medicine, Cornell University, New York, NY 10065, USA; 2 Department of Computer Science and Engineering, Islamic University and Technology (IUT), Gazipur 1704, Bangladesh; rafsanjany@iut-dhaka.edu (R.J.); azam@iut-dhaka.edu (M.A.H.); 3 Department of Mathematics and Statistics, Al-Imam Muhammad Ibn Saud Islamic University (IMSIU), Riyadh 13318, Saudi Arabia; kazad@imamu.edu.sa (A.A.); saalyami@imamu.edu.sa (S.A.A.); 4 Sewon Intelligence Ltd., Seoul 04512, Republic of Korea; sejinpark243@gmail.com; 5 Department of Robotics and Mechatronics Engineering, University of Dhaka, Dhaka 1000, Bangladesh; mmhasan@du.ac.bd

**Keywords:** eXplainable AI, electroencephalography, activity recognition, machine-learning, LIME

## Abstract

Electroencephalography (EEG) is a non-invasive method employed to discern human behaviors by monitoring the neurological responses during cognitive and motor tasks. Machine learning (ML) represents a promising tool for the recognition of human activities (HAR), and eXplainable artificial intelligence (XAI) can elucidate the role of EEG features in ML-based HAR models. The primary objective of this investigation is to investigate the feasibility of an EEG-based ML model for categorizing everyday activities, such as resting, motor, and cognitive tasks, and interpreting models clinically through XAI techniques to explicate the EEG features that contribute the most to different HAR states. The study involved an examination of 75 healthy individuals with no prior diagnosis of neurological disorders. EEG recordings were obtained during the resting state, as well as two motor control states (walking and working tasks), and a cognition state (reading task). Electrodes were placed in specific regions of the brain, including the frontal, central, temporal, and occipital lobes (Fz, C1, C2, T7, T8, Oz). Several ML models were trained using EEG data for activity recognition and LIME (Local Interpretable Model-Agnostic Explanations) was employed for interpreting clinically the most influential EEG spectral features in HAR models. The classification results of the HAR models, particularly the Random Forest and Gradient Boosting models, demonstrated outstanding performances in distinguishing the analyzed human activities. The ML models exhibited alignment with EEG spectral bands in the recognition of human activity, a finding supported by the XAI explanations. To sum up, incorporating eXplainable Artificial Intelligence (XAI) into Human Activity Recognition (HAR) studies may improve activity monitoring for patient recovery, motor imagery, the healthcare metaverse, and clinical virtual reality settings.

## 1. Introduction

The swift progress in wearable electronics, data science, artificial intelligence (AI), metaverse, the internet of things (IoT), and similar technological advancements has led to the emergence of intelligent sensor systems that are significantly transforming human lifestyles and the associated infrastructure. Human activity recognition (HAR) is a field that revolves around the identification of human activities based on data collected from sensors and various other sources. In HAR systems, the detection of object movements relies on the utilization of accelerometers, gyroscopes, inertial measurement unit (IMU), cameras, and proximity sensors [1,2,3]. While they provide valuable motion data, they lack other types of sensory information, such as cognitive, visual, or auditory cues, which may be important for certain activity recognition tasks. Physiological sensors play a crucial role in enhancing the precision of human activity recognition (HAR) systems by capturing diverse physiological parameters like heart rate, respiration rate, and skin temperature. These sensors provide valuable additional information about an individual’s physiological state. One approach to incorporating physiological sensors into HAR involves the use of wearable devices like fitness trackers and smartwatches. These devices can continuously monitor physiological parameters and transmit the collected data to a server for thorough analysis. Additionally, various biosensors, such as electrocardiogram (ECG), offer further possibilities for capturing physiological data accurately [4], electromyography (EMG) [5], foot pressure insole [6] and electroencephalography (EEG) [7,8], have been used to recognize activities of daily living (ADLs). Multimodal wireless wearable biosensors have made it possible to create Human-Computer Interaction (HCI) systems for user interfaces that are more accessible [9,10].

The choice of activity sensor depends on the type of activity being measured and the research or clinical goals. In certain applications, such as virtual spaces or metaverses where tracking virtual activities is essential, EEG can be preferred over IMU. IMU utilization may be limited in these environments. EEG provides valuable insights into cognitive processes, emotions, and neural responses during various activities, including cognitive tasks, emotion recognition, brain-computer interfaces (BCIs), sleep monitoring, and more. Additionally, EEG can detect different mental states, such as attention, meditation, drowsiness, or engagement, which may not be directly measurable with IMU sensors.

Recent cutting-edge technologies showed the potential to enhance activity recognition outcomes and healthcare delivery using the Internet of Things (IoT), wearable technology, digital twins, and big data [11,12]. Physiological signals were utilized as effective tools for the monitoring of health and the early diagnosis of disease in real time [6,13,14,15,16]. The EEG provides a valuable alternative diagnostic tool for assessing cognitive function because it can identify changes in brain rhythms caused by activity variation [17]. HealthSOS and Big-ECG were proposed that utilize EEG and ECG, data analytics to provide stroke prognosis and monitoring of stroke patients following stroke [4,18]. Wearable gait monitoring systems for stroke patients involve the use of a portable EMG device and pressure insoles to monitor the patient’s gait or walking pattern [5,19].

Machine learning (ML) and deep learning (DL) techniques have found extensive application across various domains, encompassing activity recognition, disease diagnosis, drug discovery, personalized treatment planning, and predictive analytics. However, many ML models are considered “black boxes,” lacking interpretability and immediate understandability for healthcare professionals. To address this issue, eXplainable artificial intelligence (XAI) has emerged as an approach to enhance the interpretability and trustworthiness of ML models. XAI aids in explaining the outcomes of ML algorithms, enabling doctors and therapists to gain a better understanding of how the algorithm classifies and analyzes patient activities [20,21]. To establish trust in ML-based activity recognition using EEG, an efficient ML-based HAR model has been developed, highlighting the contributions of EEG features and leveraging visual interpretability through an XAI framework such as Local Interpretable Model-Agnostic Explanations (LIME). We hypothesized that changes in activities would be reflected in EEG spectrum, enabling ML models to recognize HAR and XAI models to interpret clinically. As per our understanding, our study marks the first occurrence of introducing EEG-derived activity identification in conjunction with LIME, an interpretable AI approach highlighting the significance of EEG features in proposed machine learning HAR models. This paper presents several key contributions, which can be summarized as follows:

- Development of machine-learning classification models capable of recognizing resting, motor, and cognitive activities using EEG spectral features.
- Utilization of the LIME method to interpret the ML activity classification models and provide visual representations of the contributions made by EEG features for clinical reasoning in the context of human activity recognition (HAR).

The remaining sections of this article are organized into four parts. Section 2 provides a detailed description of the experimental protocol, including information about the EEG datasets used, EEG pre-processing techniques employed, feature extraction methods, and the machine learning (ML) approaches utilized in conjunction with the XAI approach. Section 3 presents the results of the activity prediction process, along with the insights provided by XAI techniques. Section 4 is dedicated to the discussion of the results. Finally, Section 5 presents the conclusions drawn from the research.

## 2. Materials and Methods

### 2.1. Study Design

This research study was conducted in accordance with the approved protocol by the Institutional Review Board of the Korea Research Institute of Standards and Science, located in Daejeon, South Korea. The study involved a total of 75 healthy adults with an average age of 77 years, of which 69% were female. To minimize age-related variations in physiological signals, participants were selected from a similar age group. All participants had no known history of neurological disorders. Prior to the commencement of the study, the participants were thoroughly informed about the experimental protocol. The study encompassed various activities of daily living (ADLs), including resting, motor tasks, and cognitive tasks. The motor activity tasks involved walking along a designated line and moving a bottle vertically between shelves. The resting task required the participants to lie down on a bed or recline on a sofa with their eyes closed while remaining awake. The cognitive task involved reading literature that was relatively unfamiliar to the participants. The study protocol commenced with a three-minute period of rest on the bed, followed by walking along the designated line, performing the bottle-moving task, resting on a chair, and concluding with the reading of the literature. The participants were directed to have a 5-min break after each activity interval to minimize the influence of the previous activity on the subsequent one. Throughout the entire study protocol, EEG data were continuously recorded to ensure comprehensive data collection.

### 2.2. EEG Data Acquisition

As depicted in Figure S1a, the EEG data were gathered from six specific channels, namely Fz, Oz, C1, C2, T7, and T8, using gold-plated cup electrodes in accordance with the international 10-20 EEG system. For data acquisition, a wireless Biopac BioNomadix EEG device connected to the Biopac MP 160 Module (as shown in Figure S1b) was utilized in this study. The ground electrode was placed at the FpZ point, while a reference electrode was positioned at A1, adhering to the 10-20 EEG system. To eliminate muscle tone and eye-blink artifacts, a single-channel chin electromyogram (EMG) and a single-channel electrooculogram (EOG) were recorded. Before the EEG recordings, participants were instructed to refrain from consuming coffee or alcohol. EEG data were captured during cognitive reading activities, the resting state, and motor activities such as walking and working tasks, as illustrated in Figure S1d.

### 2.3. Pre-Processing

**Figure 1:**
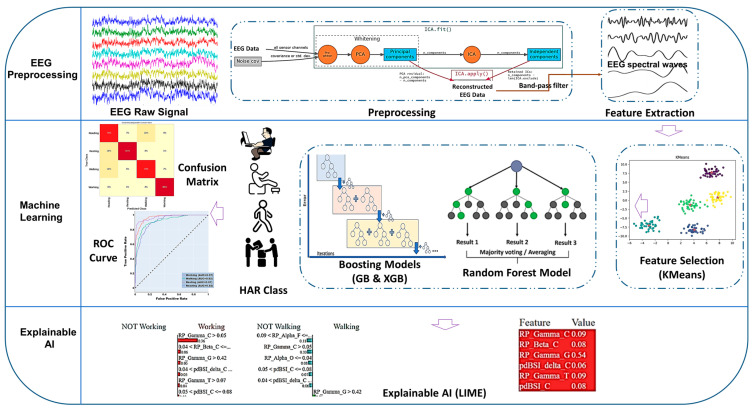
System architecture of EEG-based activity recognition approach using the eXplainable AI technique.

EEG becomes contaminated with various artifacts such as powerline noise, muscular activity, ocular activity, motion artifacts, and cardiac activity, and these artifacts remain in the raw EEG data [7,22]. The recorded EEG data was initially band-stop filtered to eliminate 60 Hz AC noise. Independent Component Analysis. (ICA) is useful to identify the individual components that correspond to the EMG, ECG, and EOG signals and can then remove them from the contaminated EEG signals, leaving only the pure EEG signal. In this study, FastICA method was used to separate unwanted artifacts from the EEG signals, shown in Figure 1 [23]. In order to filter motion artifacts, a signal-to-noise ratio (SNR) was estimated for EEG signal and the EEG epochs with SNR below 25 dB were removed from the dataset [24]. The EEG waveform was then filtered within the frequency range of 0.5–44 Hz using a band-pass filter to obtain the desired EEG spectrum. EEG data preprocessing and feature extraction were conducted using AcqKnowledge version 5.0 (Biopac Systems Inc., Goleta, CA, USA) and MNE-python [25].

### 2.4. EEG Feature Extraction

The Welch periodogram estimation method was utilized to extract EEG frequency-specific waveforms like delta, theta, alpha, beta, and gamma waves, with frequency ranges of 0.5–4 Hz, 4–8 Hz, 8–13 Hz, 13–30 Hz, and 30–44 Hz from artifact-free EEG signal [26]. By computing the power spectral density (PSD) using a 10 percent hamming window, the Fast Fourier Transforms (FFT) approach was used to extract the frequency-band waveforms of the EEG signal. Then, different features were extracted over a range of frequency ranges from the spectral waveforms that had been split into 10-s epochs. Those EEG features included the mean relative power, pairwise-derived brain symmetry index (pdBSI), delta-alpha ratio (DAR), delta-theta ratio (DTR), and (delta + theta)/(alpha + beta) ratio (DTABR) providing insight into the frequency characteristics of the EEG spectral waveforms [7]. To get the relative EEG band power, absolute EEG band powers were normalized relative to total EEG power of the entire frequency range of 0.5–44 Hz. The relative EEG band power was defined by Equation (1).

(1) $$\mathrm{RP}\_j\_k=\frac{P_{j}}{\sum_{j=1}^{q}P_{j}}$$

where, Pj represents the absolute power density of the spectral frequency at j (where j = 1, 2, …, q) and q represents the frequency range of 0.5–4 Hz, 4–8 Hz, 8–13 Hz, 13–30 Hz, and 30–44 Hz. k is the EEG electrode positions, such as frontal (F), central (C), temporal (T), and occipital (O) cortex. Features of C1 and C2 channels are averaged and refer to the central lobe (C); T7 and T8 channels are averaged and refer to the temporal lobe (T) [7].

pdBSI is calculated utilizing the method proposed by Sheoralpanday [27] and Van putten [28]. This index ranges from 0 (no asymmetry) to 1 (total asymmetry). Equation (2) defines the pdBSI.

(2) $$\mathrm{pdBSI}=\frac{1}{\mathrm{pq}}\sum_{j=1}^{q}\sum_{i=1}^{p}\left|\frac{{\mathrm{Rt}}_{\mathrm{ij}}-{\;\mathrm{Lt}}_{\mathrm{ij}}}{{\mathrm{Rt}}_{\mathrm{ij}}+{\;\mathrm{Lt}}_{\mathrm{ij}}}\right|$$

where Rt_ij_ and Lt_ij_ are the PSD of right and left channels of homologous EEG pairs (with i = 1, 2, …, p) at frequency j (with j = 1, 2, …, q).

DTR is the ratio of the power of EEG delta band to that of theta band. DTABR is the sum of the EEG power of slow waves (delta and theta) relative to that of fast waves (alpha and beta). DAR is the ratio of the power of delta band to that of alpha band [22].

### 2.5. Feature Selection

Feature selection, the process of reducing features, is vital in analyzing high-dimensional biomedical data. The accuracy of classification depends on the relevance of the features, and redundant features can cause adverse impact on computational time. SelectKBest, a Scikit-Learn library for feature selection technique, was utilized here [29]. The significance of the predictor is measured by the k-highest scores. The top 20 EEG features with higher k values were chosen for training the ML algorithms for activity recognition.

### 2.6. Machine Learning Classification

In this study, we employed machine learning techniques to classify human activities based on neurological responses captured in EEG data. Specifically, we utilized Random Forest (RF), Gradient Boosting Model (GBoost), and Extreme Gradient Boosting Model (XGBoost) algorithms to recognize four distinct human activities using EEG features.

**Figure 2:**
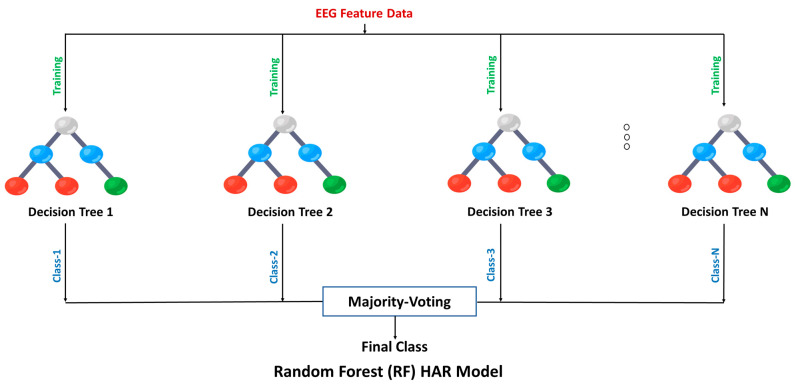
Flow chart of Random Forest Model.

**Figure 3:**
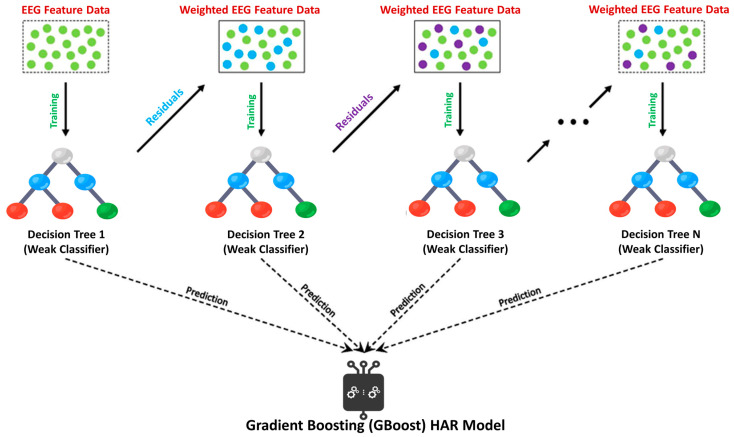
Flow chart of GBoost Model.

**Figure 4:**
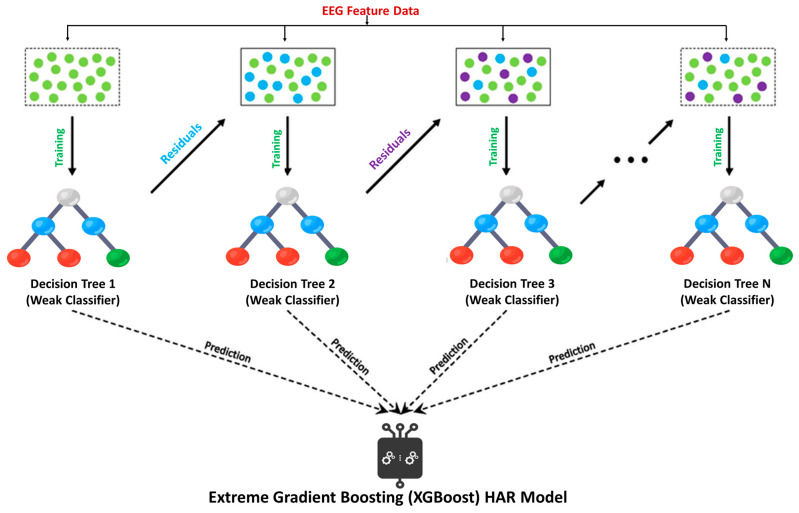
Flow chart of XGBoost Model.

RF is one of the widely used ensemble learning classifiers in machine learning studies, building a large number of decision trees, as shown in Figure 2 [30]. On the other hand, boosting algorithms use an iterative procedure to combine weak learners into strong learners [31]. GBoost is a boosting classification approach, iteratively adding decision trees to build a model, as shown in Figure 3 [32]. Another ensemble model based on Gradient Boosting with a high degree of scalability is called XGBoost, as shown in Figure 4 [33]. XGBoost constructs a loss function that is minimized if the objective of the function is expanded additively, like GBoost.

We performed non-exhaustive k-fold (k = 10) cross-validation using the training dataset to get rid of overfitting [34]. For our classification, we used the Scikit-Learn library, which offered a range of functionalities, including Decision Tree and Random Forest classifiers, and other methods [29]. Seaborn and Matplotlib libraries were utilized for the visualization of our results [35,36]. We derived various performance parameters from the confusion matrix of the ML models, such as precision, recall, F1-score, accuracy (ACC), and area under the curve (AUC). The formulae used to calculate the performance evaluation metrics are given in Equations (3)–(6).

(3) $$\mathrm{Precision}=\frac{\mathrm{TP}}{\mathrm{TP}\;+\;\mathrm{FP}}$$

(4) $$\mathrm{Recall}=\frac{\mathrm{TP}}{\mathrm{TP}\;+\;\mathrm{FN}}$$

(5) $$\mathrm{Accuracy}\left(\mathrm{ACC}\right)=\frac{\mathrm{TN}\;+\;\mathrm{TP}}{\mathrm{TN}\;+\;\mathrm{TP}\;+\;\mathrm{FN}\;+\;\mathrm{FP}}$$

(6) $$F1-\mathrm{score}=2\;*\frac{\left(\mathrm{Precision}\;*\;\mathrm{Recall}\right)}{\left(\mathrm{Precision}\;+\;\mathrm{Recall}\right)}$$

where TP denotes the true positive, TN stands for the true negative, FP represents the false positive, and FN denotes the false negative. Our experiments were conducted on Google Colaboratory, which provided us with 16 GB of RAM and a 2-core Intel Xeon Processor.

### 2.7. eXplainable Artificial Intelligence (XAI)

LIME is an open-source XAI framework that can provide interpretability to the decision-making steps of black-box ML models [37]. LIME defines the model explanation by the following formula:

(7) $$\xi \left(x\right)=\arg\underset{g\in G}{\min}L\left(f,g,\pi_{x}\right)+\Omega \left(g\right)$$

where G represents a set of interpretable models, and g denotes the complexity of the explanation, g ∈ G. Equation (7) aims to find the interpretable model ξ(x) that minimizes the sum of two terms: the loss term L(f, g, π_x), which ensures the fidelity of the interpretable model to the black-box model, and the complexity regularization term Ω(g), which promotes simplicity and interpretability. LIME ignores the process within the model and makes explanations absolutely on the data level. Therefore, the explainer explains predictions on tabular data by perturbing features based on the statistical properties of the training data.

## 3. Results

### 3.1. Activity Recognition Model Using Machine Learning Approach

As indicated in Table S1, we selected the top 20 EEG features with higher k values using feature selection to train the machine learning Human Activity Recognition models. State-of-the-art machine learning algorithms were employed to classify the human activities of healthy individuals based on EEG characteristics. Among the various ML models tested, Gradient Boosting Model (GBM) demonstrated superior performance in recognizing activities. To visualize the classification accuracy, Receiver Operator Characteristics (ROC) curves were plotted. The EEG feature dataset comprises 793 instances of reading, 408 instances of walking, 267 instances of working, and 243 instances of resting data. To train the model, 80% of the EEG feature data was used as the training dataset, and the remaining 20% as the test dataset. Figure 3 presents an example of a ROC curve for an ML model.

#### 3.1.1. Hyperparameter Tuning

To improve the ML model’s performance, several hyperparameters can be tuned, such as number of estimators, maximum tree depth, and the number of trees in the ensemble. We adjusted the hyperparameters of RF classifier model to cover the ranges of 1 to 100 for the n estimator and 1 to 30 for the max depth. RF model accuracy reached 80.33% using n estimators = 98 and max depth = 21. The accuracy using the best depth and the default value of n estimators for the GBoost classifier was not quite acceptable. The model was tuned at the parameters, and the optimal depth had a range of 1 to 14 and n estimators with a range of 1 to 50 with an interval of 1. The most acceptable result was obtained for n estimators = 50 and best dept = 8. In this hyperparameter setting, the GBoost model’s accuracy is 78.94 percent. The optimal depth for the XGBoost model included a range of 1 to 30, and the parameters and estimators included a range of 1 to 100 with an increment of 1. For n estimators of 83 and best dept of 10, the best accuracy of this model reached 79.70%.

#### 3.1.2. ML Classification Results

It is worth mentioning that the performance of activity recognition using the random forest, GBoost, and XGBoost models exhibited considerable similarity. The classification performance parameters of the machine learning (ML) activity recognition models are summarized in Table 1, Table 2 and Table 3.

The RF classifier performs better than all other approaches. The RF model classified human activities with 81% precision, 81% recall, 81% F1-score, 80.33% accuracy, and AUC: 0.92; in contrast, the XGBoost technique classified HAR with 81% precision, 81% recall, 78% F1-score, and 79.57% accuracy. Moreover, the other boosting algorithms, such as GBoost classifier achieved comparable results in terms of evaluation metrics and gave outcomes that were equal in terms of the evaluation criteria outlined above with only a 1% performance drop in each metric.

**Figure 5:**
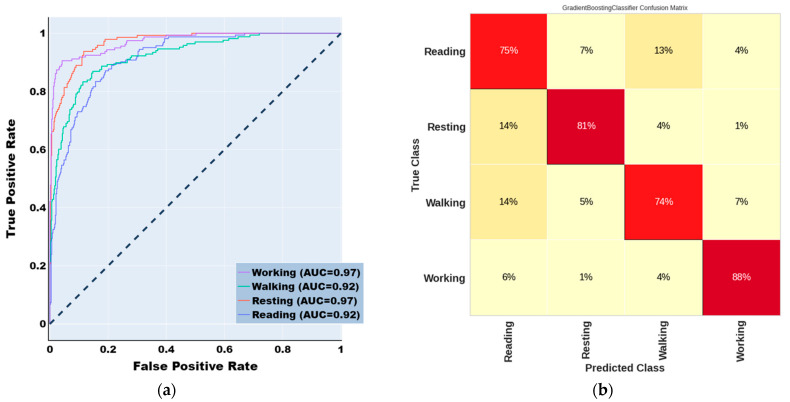
(**a**) Receiver Operating Characteristic (ROC) curves for Random Forest Model. Area under ROC curve (AUC) is an indicator of prediction accuracy. Diagonal black dotted line is the reference line. (**b**) Confusion Matrix of Random Forest in activity classification.

Overall, RF classifier was the best-performing algorithm for classifying human activities. However, all performance measurements showed that the other boosting methods competed well. The cross-validated ROC curves of individual activity classes were displayed in Figure 5. In addition, the confusion matrix of RF classifier was reported in Figure 5.

### 3.2. Interpretations of ML Models

Black box models, like the random forest, GBoost, and XGBoost ML algorithms, need to be interpreted by an XAI framework like LIME, which enables analyzing how a model predicted outcomes and explaining how various features affected those outcomes [21]. In order to establish trust in the ML model for HAR, it is essential to look at how our models predict each data instance for each class. This will enable us to decrease the feature space, which will speed up model training and improve accuracy if we can identify the key elements that influence prediction outcomes. To establish clinical trust in the ML approaches in activity prediction, it was needed to bring the role of EEG spectral features, such as gamma, delta, and theta rhythms to light. eXplainable AI may assist to investigate the contribution of EEG spectral rhythms in ML classification.

Here, the LIME model was applied in ML algorithms, such as RF Classifier to understand the prediction performance and individual role of EEG features in detecting human activities.

**Figure 6:**
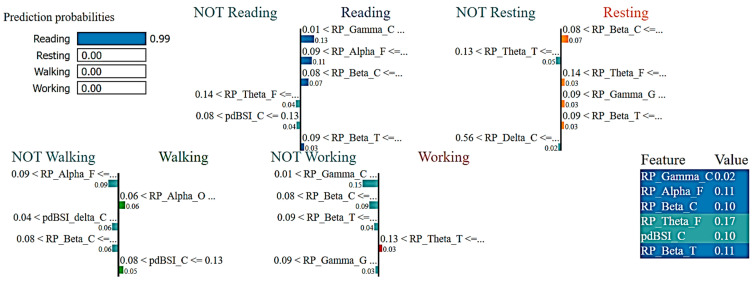
Visualization of the local contribution of EEG features through the LIME model in classifying a single test instance (predicted class = Reading activity) using the Random Forest model. The blue-marked cells represent the features that contributed most to classifying the Reading activity.

Figure 6 reports the LIME visualization for the RF Classifier to forecast a reading activity instance. The predicted probability of an instance of the reading activity is 99%. The three most contributing features of this model are RP_Gamma_C, RP_Alpha_F, and RP_Beta_C to predict a reading activity. These three features are ranked in order of importance as follows: 13%, 9%, and 8%. Using the RF Classifier, lower central gamma, lower frontal alpha, and lower frontal beta rhythms contribute more to the prediction of cognitive reading activity.

**Figure 7:**
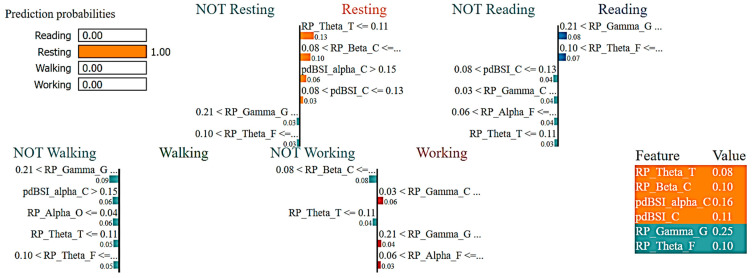
Visualization of the local contribution of EEG features through the LIME model in classifying a single test instance (predicted class = Resting activity) using the Random Forest model. The orange-marked cells represent the features that contributed most to classifying the Resting activity.

Figure 7 reports the LIME visualization for the RF Classifier to forecast a resting activity instance. The predicted probability of an instance of resting activity is 100%. For recognizing an instance of the resting activity, the three most contributing features of this model are RP_Theta_T, RP_Beta_C, and pdBSI_Alpha_C. These three features are ranked in order of importance as follows: 13%, 10%, and 6%. Using the RF Classifier, lower temporal theta, lower central beta, higher central alpha waves, and higher pdBSI have significantly greater impacts on the prediction of the resting activity.

**Figure 8:**
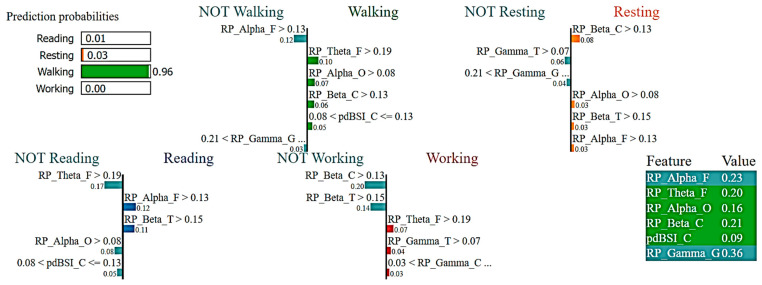
Visualization of the local contribution of EEG features through the LIME model in classifying a single test instance (predicted class = Walking activity) using the Random Forest model. The green-marked cells represent the features that contributed most to classifying the Walking activity.

Figure 8 reports the LIME visualization used by the RF Classifier to forecast a motor activity, in this case, walking. The predicted probability of the test instance of the walking activity is 96%. To predict a walking activity, the most contributing features of this model are RP_Theta_F, RP_Alpha_O, RP_Beta_C, and pdBSI_C. The feature importance of these three features are 10%, 7%, 6%, and 5% respectively. Higher frontal theta, higher occipital alpha, and higher central beta bands have much greater contributions to the prediction of motor activity using the RF Classifier.

**Figure 9:**
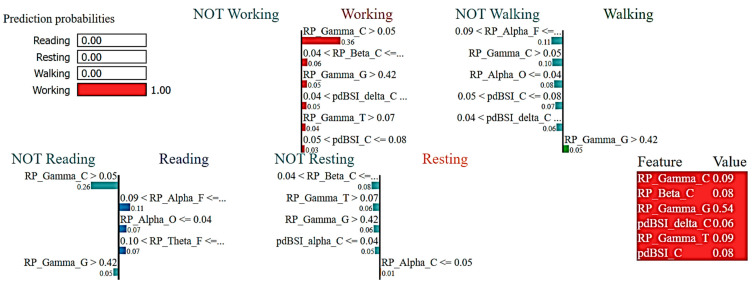
Visualization of the local contribution of EEG features through the LIME model in classifying a single test instance (predicted class = Working activity) using the Random Forest model. The red-marked cells represent the features that contributed most to classifying the Working activity.

Figure 9 reports the LIME visualization for the RF Classifier to forecast a specific instance of the working activity. The predicted probability of an instance of the working activity is 100%. To predict a working activity, topmost contributing features of this model are RP_Gamma_C, RP_Beta_C, and RP_Gamma_G. These three features are ranked in order of importance as follows: 36%, 6%, and 5%. Utilizing the RF Classifier, higher central and global gamma, and lower central beta rhythms have much greater contributions to predict motor activity.

## 4. Discussion

Our goal in this study was to interpret the activity prediction ML model using EEG data in ADLs. Specific EEG spectral power is associated with the specific functional outcome of the brain according to the demand of cognitive and motor workload in different activities. This work employs decision tree models to classify the resting, motor activities (walking and working), and cognitive reading states. LIME interpretable model described the role of EEG spectral bands for classifying activities using ML models and provides the findings of model explanation. Most earlier investigations have supported the EEG feature contribution trend provided by LIME, which has continued across all classifiers employed in this study.

In this study, the focus was on human activities involving both physical and mental tasks, which are integral to our daily routines. The three common sensory technologies used for Human Activity Recognition (HAR) were smart wearable devices with IMU sensors, cameras, and biosensors [3,9,10]. Among these, the IMU and camera primarily captured resting and physical movements, lacking the ability to detect cognitive activities. To implement HAR accurately, multiple IMU sensors were placed on various limbs of the body [3]. To overcome this limitation, innovative approaches were introduced using EEG wearable devices for HAR [8,10]. This technique has demonstrated its effectiveness in identifying both resting and cognitive activities, overcoming the limitations of previously discussed sensory technologies. However, the main drawback in previous EEG studies lies in the interpretability of ML models used.

The EEG spectrum captures variations in neurological responses that occur in response to changes in activity. Alpha wave is typically a maker of a relaxed, awake state, and has been associated with creativity, problem-solving, and other cognitive processes. EEG Alpha rhythm is also used in various fields such as cognitive research and neurofeedback [38]. Beta waves are also indicators of heightened awareness, focused attention, reading, or active problem-solving [39]. Gamma rhythm is considered to be a marker of the brain’s highest level of cognitive processing; heightened cognitive functions, including perception, memory, pattern recognition, critical problem-solving, information integration, and decision-making [40].

The brain activity associated with cognition includes visual processing, orthographic processing, phonological processing, semantic processing, and syntactic processing [41]. Reading is a cognitive task that involves the decoding and comprehension of written or printed text. The process of reading is associated with complex neural activity that involves multiple regions of the brain, including the occipital, temporal, and parietal lobes [42]. Each stage of reading is associated with different patterns of brain activity, which can be reflected in the EEG signal. EEG studies of reading have also found that different types of reading tasks, such as reading for comprehension or reading for decoding, are associated with different patterns of brain activity. For example, reading for literature is associated with a greater degree of semantic processing, while reading for decoding is associated with a greater degree of phonological processing. For example, during the initial stage of visual processing, there may be an increase in alpha and beta activity in the occipital regions of the brain, while during the later stages of semantic and syntactic processing, there may be an increase in gamma activity in the temporal and parietal regions of the brain [43].

Lower temporal theta, lower central beta, and higher central alpha is observed in the resting state EEG. Alpha plays a role in attention regulation and sensory processing; theta is thought to play a part in memory consolidation and attention regulation [44]. Reduced theta, beta activity, and increased alpha best describe the resting state [7]. The working activity, the object moving task, is associated with different stages of motor planning and control, such as the guided reaching and grasping movements. Gamma activity is increased in the primary motor cortex during the planning and execution of reaching and grasping movements [45]. Additionally, studies have found that gamma activity is modulated by the difficulty of a motor task, with higher levels of gamma activity observed during more difficult tasks [46].

Motor tasks are a complex process, associated with different cognitive and motor functions, and it is related to different EEG bands. Alpha activity is typically observed in the occipital and parietal regions during walking, and it is thought to be related to visual perception and spatial attention. Beta activity is typically observed in the primary motor cortex during walking, and it is thought to be related to motor planning and control, attention, and perception [47]. Gamma activity, observed in the primary motor cortex during walking, is thought to be related to the integration of sensory information and motor planning [48]. Additionally, the EEG activity in the delta band may also increase in the sensorimotor cortex. This could be associated with the repetitive and rhythmic nature of the walking.

In collaborative platforms, like smart home, cyber metaverse, human-robot interaction, and autonomous transportation, it is expected that people can interact with each other in a shared virtual environment. HAR can facilitate understanding human activities within a collaborative environment, performing specific cognitive tasks within the virtual environment, personalized healthcare monitoring, elderly assisted living, and collaborative robots. It is evident that activity recognition is a challenging cognitive job involving several different brain regions and each EEG signal could reflect only a part of the neural response involved in an activity. Therefore, it’s important to use high-density EEG to have a comprehensive understanding of ADLs. Although we used clinical EEG in this study to explore changes in EEG caused by tasks or human activities, consumer-grade EEG may facilitate HAR in real-life scenarios. In forthcoming times, we plan to expand our research by conducting cross-laboratory experiments to automate activity recognition utilizing multimodal wearable biosensors.

## 5. Conclusions

The rapid advancement of the cyber metaverse has opened up exciting possibilities in the field of human activity recognition research. In this study, machine learning techniques were employed to recognize human activities using clinical EEG data. To enhance interpretability, eXplainable artificial intelligence techniques were successfully integrated to provide clinical reasoning behind the EEG spectral features in human activity recognition (HAR). The results of this research using eXplainable AI in HAR have significant potential in rehabilitation settings. Therapists can leverage this information to customize treatment plans according to the specific needs and capabilities of individual patients. This advancement holds great promise in improving rehabilitation outcomes, virtual reality, and optimizing patient care.

## Figures and Tables

**Table 1:** Results of the Classification performance of Random Forest Classifier.

Random Forest Classifier (Accuracy = 80.33%)			
Activity Class	Precision	Recall	F1-Score
Reading	0.77	0.71	0.74
Resting	0.78	0.88	0.82
Walking	0.78	0.82	0.80
Working	0.89	0.82	0.86
**Weighted Average**	**0.81**	**0.80**	**0.80**

**Table 2:** Results of the Classification performance of Extreme Gradient Boosting Classifier.

XGB Classifier (Accuracy = 79.70%)			
Activity Class	Precision	Recall	F1-Score
Reading	0.78	0.7	0.74
Resting	0.78	0.89	0.83
Walking	0.76	0.77	0.76
Working	0.88	0.85	0.86
**Weighted Average**	**0.80**	**0.80**	**0.80**

**Table 3:** Results of the Classification performance of Gradient Boosting Classifier.

Gradient Boosting Classifier (Accuracy = 78.94%)			
Activity Class	Precision	Recall	F1-Score
Reading	0.75	0.7	0.73
Resting	0.76	0.87	0.81
Walking	0.75	0.79	0.77
Working	0.9	0.81	0.86
**Weighted Average**	**0.79**	**0.79**	**0.79**

## Data Availability

Not applicable.

## Supplementary Materials

The following supporting information can be downloaded at: https://www.mdpi.com/article/10.3390/s23177452/s1, Figure S1: EEG Signal Processing and electrode positions and layout. (a) six-channel EEG, reference and ground electrodes position based on Standard 10-20 EEG system, (b) Biopac EEG module, (C) Experimental scenario, (c) Activity Classes with scenarios. Table S1. List of the top 20 EEG features with higher k values using feature selection to train the machine learning Human Activity Recognition (HAR) models. RP: Relative Power; T: Temporal lobe; C: Central lobe; F: Frontal lobe; O: Occipital lobe; G: Global (Averaged over F, C, T, and O).
