# Supplementary material for: An Explainable EEG-Based Human Activity Recognition Model Using Machine-Learning Approach and LIME
Source: Sensors (Basel). 2023 Aug 27;23(17):7452. doi: 10.3390/s23177452 (PMC10490625; doi:10.3390/s23177452)
Supplement: Supplementary file 1 [file sensors-23-07452-s001.zip › sensors-2528089-supplementary.pdf]

Supplementary Section

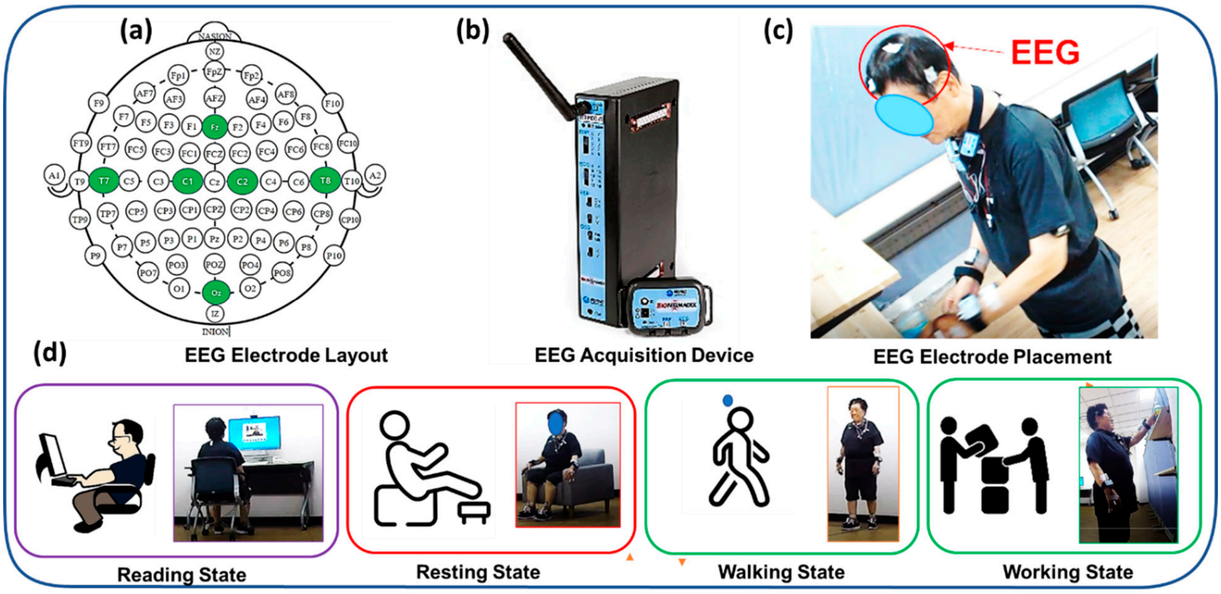

Figure S1. EEG Signal Processing and electrode positions and layout. (a) six-channel EEG, reference and ground electrodes position based on Standard 10–20 EEG system, (b) Biopac EEG module, (C) Experimental scenario, (c) Activity Classes with scenarios.

Table S1. List of the top 20 EEG features with higher k values using feature selection to train the machine learning Human Activity Recognition (HAR) models. RP: Relative Power; T: Temporal lobe; C: Central lobe; F: Frontal lobe; O: Occipital lobe; G: Global (Averaged over F, C, T, and O).

1. RP_Theta_T
2. RP_Theta_G
3. RP_Gamma_C
4. RP_Beta_F
5. RP_Beta_G
6. RP_Theta_O
7. RP_Theta_C
8. RP_Beta_T
9. RP_Gamma_G
10. RP_Theta_F
11. RP_Beta_C
12. RP_Alpha_F
13. pdBSI_alpha_C
14. RP_Gamma_T
15. RP_Gamma_O
16. pdBSI_C
17. pdBSI_gamma_C
18. pdBSI_beta_C
19. RP_Beta_O
20. RP_Alpha_G
